# Parenting Stress in Adolescent Anorexia Nervosa: Differential Stress in Mothers and Fathers During Inpatient Treatment

**DOI:** 10.1002/eat.24551

**Published:** 2025-09-18

**Authors:** V. Stonawski, J. Kutzner, S. Büscher, A. Hoyer, O. Kratz, S. Horndasch

**Affiliations:** ^1^ Department of Child and Adolescent Mental Health University Hospital Erlangen, Friedrich‐Alexander University Erlangen‐Nürnberg (FAU) Erlangen Germany; ^2^ Biostatistics and Medical Biometry Medical School OWL, Bielefeld University Bielefeld Germany; ^3^ Department of Child and Adolescent Psychiatry and Psychotherapy Bielefeld University, Medical School and University Medical Center OWL, Protestant Hospital of Bethel Foundation Bielefeld Germany

**Keywords:** adolescence, anorexia nervosa, fathers, mothers, parenting stress

## Abstract

**Objective:**

Anorexia nervosa (AN) is a severe psychiatric disorder that impacts both the adolescents themselves and their parents. Parenting stress is associated with impaired mental health of parents and poorer treatment outcomes for their children. Understanding the course of parenting stress during inpatient treatment and differences between mothers and fathers is important to improve AN treatment and (long term) outcome.

**Method:**

Within a longitudinal controlled design, parents of adolescents with AN are compared to healthy children (HC) at three time points during and after their daughters' inpatient treatment (T1 = admission, T2 = discharge/+4 months, T3 = 6‐month follow‐up) regarding parenting stress. Mothers (T1: AN *n* = 29, HC *n* = 25; T3 (after dropout): AN *n* = 11, HC *n* = 18) and fathers (T1: AN *n* = 25, HC *n* = 22; T3 (after dropout): AN *n* = 9, HC *n* = 13) rated their parenting stress via the German version of the Parenting Stress Index (PSI) and their own psychological symptoms via the Brief Symptom Inventory (BSI). Diverse individual, adolescent, AN‐ and treatment‐associated characteristics were assessed and related to parenting stress.

**Results:**

Within the AN group, particularly at admission, parenting stress of mothers was higher compared to fathers. Mothers of adolescents with AN had higher, while fathers of adolescents with AN had lower parenting stress scores compared to those of HC at inpatient admission. During inpatient treatment, parenting stress decreased in mothers and increased in fathers of adolescents with AN, resulting in no differences after inpatient treatment. Comparing mothers and fathers directly showed higher scores in mothers compared to fathers in the AN group. Parental psychological symptoms predicted parenting stress, as opposed to child or treatment associated factors.

**Discussion:**

Parenting stress and the own psychological symptoms of parents should be considered explicitly in inpatient treatment of adolescents with AN. Furthermore, differences between mothers and fathers should be considered in future research and parent‐focused interventions.

**Summary**

- Adolescent Anorexia nervosa impacts both the adolescents themselves and the parents.
- The consideration of parenting stress and parental psychological symptoms in the treatment of adolescents with AN is essential.
- Differences between mothers and fathers should be specifically addressed in parent‐focused interventions.

## 1 Introduction

Anorexia nervosa (AN) is a severe psychiatric disorder with multiple somatic complications, a high risk of becoming chronic, and an increased risk of mortality, which typically starts in adolescence (Filipponi et al. 2022; Katzman 2005; Quadflieg et al. 2024; Søeby et al. 2024; Steinhausen et al. 2008). Thereby, AN impacts not only the adolescents themselves but also their parents and families (McCormack and McCann 2015). Worse family functioning was found in families with adolescent eating disorders (ED), with more pronounced ED symptoms being associated with worse family functioning (Holtom‐Viesel and Allan 2014; Rienecke et al. 2024). Thus, the involvement of parents or families is a highly relevant component of the treatment of AN in children and adolescents (Frostad and Bentz 2022; Herpertz et al. 2018).

Parenting is a multifaceted challenge itself (Nomaguchi and Milkie 2020), but raising and supporting children and adolescents with mental disorders was found to be particularly challenging and stressful (Zeng et al. 2023). The construct of parenting stress refers to specific stressors associated with the parental role that appear to exceed the parent's own available resources and can be divided into two dimensions: stress related to the parent's own demands and the child's demands (Abidin 1995; Ríos et al. 2022). Thereby, high parenting stress was associated bidirectionally with child behavior problems (Barroso et al. 2018; Neece et al. 2012). Looking at clinical cohorts, caregiving burden was highest in caregivers of patients with EDs compared to those of patients with depression or schizophrenia (Martín et al. 2015). Within EDs, a restrictive ED like AN was associated with higher parental stress compared to others (Graap et al. 2008; Orive et al. 2013), with parental roles and tasks being very diverse here: Before treatment, parents are challenged by for example, recognizing the severe illness, dealing with acute symptoms, and seeking support. During treatment, they are then typically used as “co‐therapists” and responsible for implementing interventions at home, providing meals, motivating the child to eat or interrupting dysfunctional symptoms, and providing emotional support and guidance (Kyriacou et al. 2008; Martín et al. 2015; Stillar et al. 2022). In addition, a potentially necessary inpatient treatment of the child was shown to elicit high stress in parents (Commodari 2010; Franck et al. 2015). Overall, parents of adolescents with AN showed a generally high level of burden and emotional distress as well as impairments regarding psychological health, physical health, and romantic relationships (Duclos et al. 2023; Irish et al. 2024; Kyriacou et al. 2008; Martín et al. 2015; Ohara et al. 2016; Wilksch 2023). Parenting stress and psychological symptoms were found to be associated bidirectionally and may interfere with each other (Cohen 2000; Duclos et al. 2023; McCormack and McCann 2015). Both more parental distress and emotional symptoms, in turn, were associated with lower confidence in the parent's own competencies, lower self‐efficacy, and dysfunctional caregiving styles (Konstantellou et al. 2021; Monteleone et al. 2021; Stillar et al. 2022). Moreover, negative associations with the patient's disorder and treatment were found (Monteleone et al. 2021; Timko et al. 2023).

Various parent, child, and situational factors proved to be relevant for stress in parents of healthy children, as for example, parental depressive symptoms, child overall problems, social support, or the educational level (Fang et al. 2024). Among parents of children with AN, some studies could identify additional disorder‐specific factors relevant for stress, as for example, a more severe AN, a lower premorbid weight, or a shorter treatment duration (Anastasiadou et al. 2014; Duclos et al. 2023; Kramer et al. 2023). In contrast, other studies found the parental subjective perspective of the disorder to be more relevant for parental stress than objective indicators of AN severity (Matthews et al. 2018; Ohara et al. 2016; Padierna et al. 2013). Moreover, individual parental factors, as for example, parental psychological symptoms, a personal history of ED, or a strong intolerance of uncertainty, and social conditions, as for example, being the primary carer or divorced, appeared to be relevant (Duclos et al. 2023; Konstantellou et al. 2021; Kramer et al. 2023; Padierna et al. 2013; Stefanini et al. 2019; Timko et al. 2023).

Former results hint at differences between mothers and fathers, with studies reporting a higher impairment in mothers: Mothers were found to have a higher caregiving burden, more psychological distress as well as stronger fears regarding involvement in the child's treatment (Anastasiadou et al. 2014; Kyriacou et al. 2008; Martín et al. 2015; Raenker et al. 2013; Stillar et al. 2022). Fathers experienced themselves more as secondary support compared to the mothers' role as the main responsible party during the child's treatment process (O'Sullivan et al. 2024) and reported lower ED‐related burden and emotional overinvolvement compared to mothers (Anastasiadou et al. 2014; Zeiler et al. 2023). Significant, but inconsistent differences between mothers and fathers were observed regarding the factors that influence their stress levels (e.g., Duclos et al. 2023; Stillar et al. 2022).

While cross‐sectional studies have shown a higher level of stress in parents of adolescents with AN, to the best of the authors' knowledge, hardly any study has investigated this across inpatient treatment, especially for mothers and fathers separately. We hypothesized (1) a higher parenting stress in mothers and fathers of hospitalized adolescents with AN compared to those of healthy children (HC) and (2) a decline in parenting stress during and after inpatient treatment. In exploratory analyses, we compared parenting stress (3) between mothers and fathers of adolescents with AN. Finally, (4) we hypothesized parenting stress to be associated with individual (age, relationship status, psychological distress), child‐associated (age, psychopathology, BMI) and treatment‐associated (treatment duration, symptom change) factors.

## 2 Method

### 2.1 Study Design

Current data were collected within the larger basis module of the FRAnconian Longitudinal study of Anorexia Nervosa in Adolescents (FRALANA), which follows the course of inpatient treatment of adolescents with AN using a multi‐level approach. The current inpatient treatment on an ED‐focused ward has a cognitive‐behavioral orientation and includes regular involvement of the adolescents' parents: therapist‐parent or therapist‐family appointments including for example, psychoeducation and parent guidance on dealing with the illness (at least 2×/month); an ED‐specific parent group (1×/month); supervised family meals; and up to 4 visits per week, which gradually increase in duration up to therapeutic day or weekend leaves, which include more and more meals together at home or outside and evaluation afterwards with the therapist afterwards. In a controlled longitudinal study design, patients with AN and their parents were compared to HC at three time points (T1, admission; T2, discharge/+4–5 months; T3, 6‐month follow‐up after discharge/T2). So total study time was around 10–11 months for each family (for the AN‐group in dependence of treatment duration). Data of both groups were collected in parallel between 11/2020 and 02/2024. The current study is a secondary analysis of the basic module of the FRALANA study. Ethical approval for the FRALANA study was granted by the local ethics committee. The study was prospectively registered with the German Clinical Trials Register (ID number DRKS00024752) and conducted in accordance with the Declaration of Helsinki.

### 2.2 Study Population

Female adolescents between 10 and 17 years of age were included in this study. For the AN group, adolescents had to be diagnosed with AN according to ICD‐10 criteria by an experienced (child) psychiatrist or psychologist using the “Kinder‐DIPS” diagnostic interview (Margraf et al. 2017; Schneider et al. 2017) and therefore treated in an inpatient child‐ and adolescent‐psychiatric setting. For HC, a weight corresponding to a Body Mass Index (BMI) below the 10th age percentile and any psychiatric disorder (currently or in history) was an exclusion criterion. General exclusion criteria included acute psychotic symptoms, substance abuse/addiction, medication with tranquilizing effects (such as Benzodiazepines), learning disabilities, or insufficient German skills to give informed consent. The recruitment of patients was conducted by trained therapists at the admission to patients' inpatient treatment. Recruitment of HC was carried out via advertisements, notices in public places, and through private contacts. After checking eligibility, adolescents and their parents were informed about the study and gave their informed consent before participating.

Within the current study, 54 mothers (AN: *n* = 29, HC: *n* = 25), 47 fathers (AN: *n* = 25, HC: *n* = 22) and 55 female adolescents (AN: *n* = 30, HC: *n* = 25) took part, including at least one parent of each adolescent. For 47 adolescents (87%; AN: *n* = 25; HC: *n* = 22), both parents took part at T1, with groups not differing from each other. A drop‐out of parents was observed across the three assessments (drop‐out rates: 26% to T2, further 22% to T3), with higher drop‐out rates in the AN compared to the HC group, and in total, parents of 28 adolescents completed all three assessments (AN: *n* = 10, 34%; HC: *n* = 18, 72%). Completing and dropping‐out parents did not differ in terms of their age, parenting stress, or their own psychological symptoms (*t*‐tests: *p* > 0.05).

### 2.3 Measures

As individual measure, parenting stress was assessed for both mothers and fathers via the German version of the Parenting Stress Index (PSI; Abidin 1995; German version: Eltern‐Belastungs‐Inventar, EBI, Tröster 2010). Besides the total stress scale, the subscales “parent domain” (e.g., “Some aspects of raising my child are more challenging than I anticipated.” or “I have been physically exhausted for the last six months.” or “I sometimes feel restricted by my responsibilities as a mother/father.”) and “child domain” (e.g., “My child does some things that are very challenging for me.” or “I feel that my child needs more attention and care than other children.”), that represent sources of stress based on the reported child or parent characteristics, respectively, were used in analyses. To assess parental psychological symptoms, mothers and fathers rated their own symptoms via the Brief Symptom Inventory (BSI; Derogatis 1993); the Global Severity Index (GSI) was used as measure of global distress. Sociodemographic data of mothers and fathers regarding age, relationship status (living in a relationship vs. single‐parent), and working status (full‐time, part‐time, non‐working) were assessed.

As child characteristics, adolescents' height and weight was measured at each assessment, with the BMI and BMI SDS being calculated afterwards. Psychopathology was assessed via questionnaires in self‐ and parent‐report, with consistently higher values indicating a higher psychopathology: ED‐psychopathology was assessed via the self‐report Eating Disorder Inventory‐2 (EDI‐2; Paul and Thiel 2004), using the sum score of the short version and the AN‐specific subscale “drive for thinness” in analyses. The sum‐score of the Questionnaire for relatives of patients with anorexia nervosa (German: Fragebogen für Angehörige von Patienten mit Anorexia nervosa, FAPAN; Vandereycken and Meermann 2003) was used as parental rating of adolescents' AN psychopathology. Adolescents' global psychopathology was rated by mothers and fathers via the Child Behavior Checklist (CBCL; German version: Döpfner et al. 2014), with the total sum score being used for analyses.

### 2.4 Statistical Analyses

Group differences regarding for example, sociodemographic and psychopathologic characteristics in parents and adolescents were assessed descriptively. Linear mixed regression models with random intercepts accounting for repeated measurements and family membership were used to investigate the parenting stress (separately for the three PSI scores: parent domain, child domain, total stress scale) over the course of treatment between both groups (AN, HC). To examine the effects of group allocation (AN, HC), parent‐specific effects (mothers, fathers), and time effects (T1, T2, T3), as well as interaction effects among these variables, 12 dummy variables were introduced. Each dummy variable is representative of a unique combination of group, parent, and time point, rather than using an intercept term and individual covariates for these three different effects. To investigate and account for potential confounding variables, we additionally included individual, child, and treatment associated variables: the maternal or paternal psychological symptoms (GSI) due to a consistent association with the PSI scores and a difference between AN and HC mothers, as well as the parent and child age, the parental relationship status, the child weight, the EDI‐2, FAPAN and CBCL sum score as covariates. Regression coefficients with corresponding 95% confidence intervals were given as effects estimates. In order to evaluate group differences (e.g., between fathers and mothers, or between AN parents and HC parents), the general linear hypothesis *F*‐test is employed. In this instance, the null hypothesis ($H_{0}:\mathrm{C\beta}=d$) is tested against the alternative hypothesis ($H_{1}:\mathrm{C\beta}\neq d$), where the matrix C and the vector d define the linear hypothesis to be tested (Fahrmeir 2013). This enables the assessment of the equivalence of sets of parameters, and consequently facilitates the comparison of groups defined by multiple parameters. A complete case analysis was conducted, where a complete case was considered to be a time point at which information on all relevant covariates was available for the parent under consideration. For all analyses, significance level was set to *p* < 0.05. As sensitivity analysis, we fitted the model separately for mothers and fathers. In order to assess the regression outcomes concerning group‐, parent‐, and time‐specific variations in terms of model robustness, the tests were repeated on altered models, where controlling variables with non‐significant effects were removed in a backward‐selection process. All analyses were performed with the statistical software R (version 4.5.1) (R‐Core‐Team 2025) and the linear mixed models were estimated using the package lme4 (version 1.1–37; for the used R‐syntax, see Code S1) (Bates et al. 2015) and lmerTest (version 3.1–3) (Kuznetsova et al. 2017) for the calculation of the confidence intervals and the general linear hypothesis *F*‐tests (for an exemplary R‐syntax, see Code S2).

## 3 Results

### 3.1 Sample Description

The age of participating mothers ranged between 35.6 and 58.1 years. 79% stated they live in a relationship and 31%/61% work full‐ or part‐time, respectively. Mothers of adolescents with AN reported more own psychological symptoms in the BSI compared to mothers of HC. The fathers' age ranged between 41.1 and 57.3 years. 93% stated they live in a relationship and 93%/6% work full‐ or part‐time, respectively. The fathers of the AN and HC group are comparable in socio‐demographic variables and in their self‐rated psychological symptoms. Participating adolescents were between 10.5 and 17.11 years of age and comparable between groups. Within the AN group, *n* = 23 adolescents had a diagnosis of a restrictive AN, *n* = 6 of a binge‐purging subtype and *n* = 1 adolescent of an atypical AN. 43% of the adolescents with AN (*n* = 14) had at least one comorbid psychiatric diagnosis. In accordance with the group assignment, adolescents with AN had a lower BMI and more ED and general psychopathologic symptoms in self‐ and parent‐ratings at T1. They also showed a larger weight gain and reduction in ED or psychopathologic symptoms from T1 to T2. Treatment duration (T1–T2 period from admission to discharge) of adolescents with AN ranged between 5 and 46 weeks (mean: 22.5 weeks), with the control group T1–T2 period being matched to this, resulting in comparable time differences (mean: 21.7 weeks). All descriptives regarding parental, child, and treatment characteristics are depicted in Table 1.

**TABLE 1:** Descriptives of mother, father, adolescent characteristics, separated by groups.

		AN		HC	
		*N*	*M* (SD)/*n* (%)	*N*	*M* (SD)/*n* (%)
*Mothers (T1)*					
Age	in years	29	46.8 (5.1)	25	44.3 (5.3)
Relationship status	in relationship	29	23 (79%)	25	20 (80%)
Working status	full‐time	29	8 (28%)	25	9 (36%)
	part‐time		17 (58%)		16 (64%)
	non‐working		4 (14%)		0 (0%)
Psychological symptoms	BSI: GSI score	29	0.49 (0.44)	24	0.18 (0.22)
*Fathers (T1)*					
Age	in years	25	49.5 (3.6)	22	46.7 (6.9)
Relationship status	in relationship	25	23 (92%)	22	21 (95%)
Working status	full‐time	25	25 (100%)	22	19 (86%)
	part‐time		0 (0%)		3 (14%)
	Non‐working		0 (0%)		0 (0%)
Psychological symptoms	BSI: GSI score	25	0.39 (0.49)	20	0.23 (0.27)
*Adolescents*					
Age (T1)	in years	30	14.8 (1.4)	25	14.2 (2.4)
BMI	at T1 in kg/m^2^	30	14.6 (1.1)	25	19.8 (3.6)
	at T2 in kg/m^2^	28	18.4 (1.0)	22	19.9 (3.9)
BMI SDS	at T1	30	−3.37 (1.18)	25	−0.32 (0.98)
	at T2	28	−0.95 (0.30)	22	−0.38 (1.00)
ED psychopathology (T1)	EDI‐2 sum score (self)	27	220.3 (41.3)	25	153.8 (43.4)
	EDI‐2 drive for thinness (self)	28	32.3 (8.3)	25	14.7 (7.2)
	FAPAN (mother)	29	28.5 (9.4)	25	5.2 (2.4)
	FAPAN (father)	24	30.2 (8.6)	22	5.9 (6.0)
Global psycho‐pathology (T1)	CBCL sum score (mother)	29	32.2 (15.5)	25	12.7 (14.1)
	CBCL sum score (father)	24	34.5 (19.1)	20	11.3 (15.2)
*Treatment characteristics (T1‐T2)*					
Treatment duration/time T2‐T1	in weeks	28	22.5 (10.7)	22	21.1 (4.7)
Weight change	in kg	28	10.9 (3.7)	22	0.95 (1.9)
Change in adolescents' psychopathology	EDI‐2 sum score (self)	20	−19.7 (26.5)	22	−1.36 (27.7)
	EDI‐2 drive for thinness (self)	21	−7.57 (7.0)	22	−0.32 (5.1)
	FAPAN (mother)	19	−11.0 (8.8)	18	0.17 (3.7)
	FAPAN (father)	16	−17.5 (11.9)	16	0.03 (2.5)

Abbreviations: AN, anorexia nervosa group; BMI SDS, BMI standard deviation score; BSI, Brief Symptom Inventory; CBCL, Child Behavior Checklist; EDI‐2, Eating‐Disorder‐Inventory; FAPAN, Questionnaire for parents of patients with anorexia nervosa; GSI, Global Severity Index; HC, healthy children; T1, admission/first assessment; T2, discharge/second assessment; T3, 6‐month follow‐up.

### 3.2 Parenting Stress at Admission and After Treatment

**FIGURE 1:**
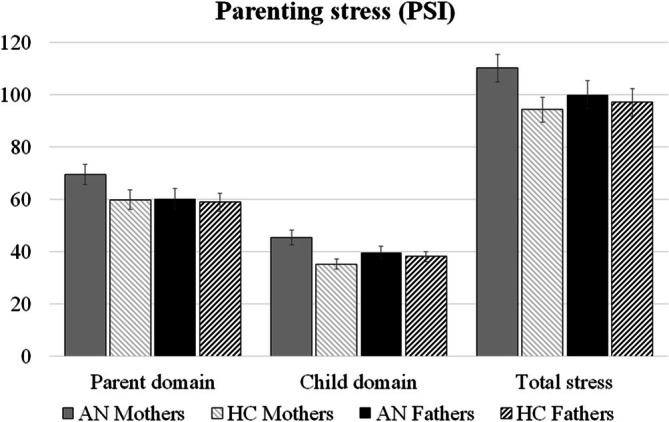
Parental stress (PSI) at admission (T1), separated for the AN and HC groups and for mothers and fathers. Mean and standard error (SE) are depicted.

Regarding the parenting stress at admission (T1), mothers of AN and HC adolescents differed regarding the parent domain, the child domain, and the total stress scale: AN mothers rated their parental stress consistently higher than HC. In contrast, fathers of AN and HC adolescents had comparable PSI scores descriptively. Full descriptive results of PSI scores can be found in Table 2 and are shown in Figure 1. Controlling for relevant covariates (GSI, parent and child age, parental relationship status, child weight, EDI‐2, FAPAN and CBCL sum score), the linear mixed regression model for both parents revealed a significant difference in the PSI child domain between AN and HC (*p* = 0.023; robustness check: *p* = 0.014). Here, a differential pattern for the parents was found: while AN mothers had higher PSI scores, AN fathers had lower PSI scores compared to their corresponding HC parents at admission.

**TABLE 2:** Descriptives of parenting stress (PSI), separated by parent and by groups.

		AN		HC	
		*n*	*M* (SD)	*n*	*M* (SD)
*PSI*	*Mothers*				
T1	Parent domain	29	69.5 (20.9)	25	59.0 (18.1)
	Child domain		45.4 (15.4)		35.3 (9.7)
	Total stress scale		110.2 (28.2)		94.3 (23.5)
T2	Parent domain	19	66.6 (21.4)	19	66.2 (16.1)
	Child domain		41.6 (12.3)		36.7 (10.2)
	Total stress scale		108.3 (29.2)		102.8 (21.0)
T3	Parent domain	11	62.9 (20.0)	18	64.2 (12.3)
	Child domain		40.7 (12.3)		40.2 (9.7)
	Total stress scale		103.6 (30.3)		101.3 (25.4)
*PSI*	*Fathers*				
T1	Parent domain	25	60.2 (20.1)	22	58.9 (16.6)
	Child domain		39.8 (10.9)		38.2 (9.2)
	Total stress scale		100.0 (27.1)		97.1 (24.2)
T2	Parent domain	15	64.7 (17.7)	16	64.1 (17.9)
	Child domain		46.5 (13.0)		35.9 (9.5)
	Total stress scale		111.1 (25.7)		99.9 (24.6)
T3	Parent domain	9	71.8 (20.6)	13	59.7 (15.1)
	Child domain		42.3 (10.7)		36.8 (11.7)
	Total stress scale		118.1 (85.5)		85.5 (28.1)

*Note*: Scores of the German version of the Parenting Stress Index (PSI) for the parent domain, child domain, and total stress scale.

Abbreviations: AN, anorexia nervosa group; HC, healthy children; T1, admission/first assessment; T2, discharge/second assessment; T3, 6‐month follow‐up.

**FIGURE 2:**
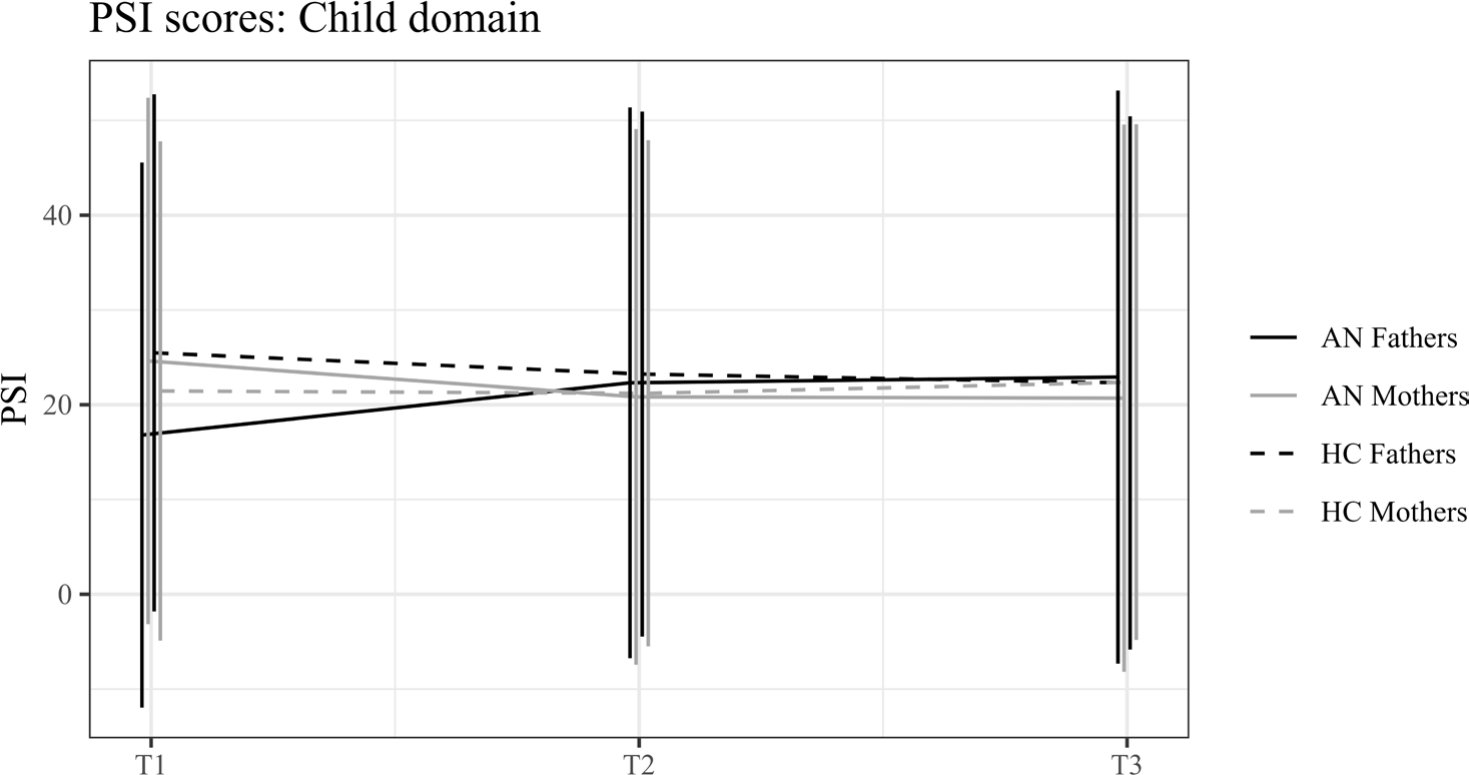
The estimated group‐specific, parent‐specific, and time‐specific intercept parameters of the main linear mixed regression model of parenting stress in the child domain (PSI) from admission (T1) to discharge (T2) and 6‐month follow‐up (T3), separated for the AN and HC groups and for mothers and fathers. Estimated coefficients and their standard deviation (SD) are depicted.

To investigate the parenting stress over the course of and after treatment, linear mixed regression models were estimated. In addition to the joint model (main analysis; for complete results, see Table 3), separate models for mothers and fathers (sensitivity analyses; for complete results, see Tables S1 and S2) and with a reduced number of controlling variables (robustness analyses; all reported group‐, parent‐, and time‐specific differences prevailed in terms of effect direction and, with the exception of differences in the AN group from T1 to T2, also in terms of statistical significance; for complete results, see Table S3) were estimated. In the LMM complete case analysis, a slightly reduced sample size was included due to missing values in covariates (mothers: T1: AN *n* = 26, HC *n* = 21; T2: AN *n* = 18, HC *n* = 14; T3: AN *n* = 8, HC *n* = 11; fathers: T1: AN *n* = 20, HC *n* = 14; T2: AN *n* = 13, HC *n* = 10; T3: AN *n* = 5, HC *n* = 8). In the HC group, the PSI scores did not differ significantly over time (neither in mothers nor in fathers); however, the main analysis revealed evidence for a significant change in the PSI child domain in the AN group over the treatment course (T1 to T2; *p* = 0.043; robustness check: *p* = 0.116). Again, differential patterns for mothers and fathers emerged: While parenting stress decreased over time in mothers, parenting stress increased in fathers of the AN group. Afterwards, no significant changes were found until the follow‐up 6 months after discharge. Thus, while differences between the groups were found at T1 (see above), the groups “converged” at T2 and no longer differed from each other (child domain: *p* = 0.983) as can be seen in Figure 2.

**TABLE 3:** Results from linear mixed regression models regarding the course of parenting stress (PSI) comparing AN to HC.

PSI	Covariate	Regression coefficient	95%‐confidence interval	*p*
Parent domain	AN Mothers T1	19.52	[−27.10; 66.13]	0.415
	AN Mothers T2	16.60	[−30.87; 64.06]	0.496
	AN Mothers T3	17.95	[−30.34; 66.24]	0.469
	AN Fathers T1	8.36	[−39.78; 56.50]	0.735
	AN Fathers T2	14.47	[−34.34; 63.27]	0.564
	AN Fathers T3	20.56	[−29.54; 70.66]	0.424
	HC Mothers T1	18.20	[−26.42; 62.82]	0.428
	HC Mothers T2	22.54	[−22.56; 67.63]	0.332
	HC Mothers T3	23.14	[−22.83; 69.12]	0.328
	HC Fathers T1	16.94	[−29.10; 62.99]	0.474
	HC Fathers T2	22.18	[−24.47; 68.82]	0.356
	HC Fathers T3	14.50	[−32.94; 61.93]	0.552
	BSI: GSI score	19.42	[8.83; 30.01]	0.001
	FAPAN	0.27	[−0.07; 0.60]	0.122
	CBCL sum score	0.07	[−0.23; 0.38]	0.635
	Age (parents)	0.25	[−0.68; 1.17]	0.603
	In relationship (yes vs. no)	10.98	[−0.15; 22.11]	0.057
	Age (child)	−1.61	[−4.41; 1.19]	0.266
	Weight	0.57	[0.06; 1.07]	0.031
	EDI‐2 sum score (self)	0.05	[−0.02; 0.12]	0.140
Child domain	AN Mothers T1	24.62	[−3.15; 52.39]	0.088
	AN Mothers T2	20.83	[−7.43; 49.08]	0.155
	AN Mothers T3	20.69	[−8.17; 49.56]	0.166
	AN Fathers T1	16.80	[−11.95; 45.56]	0.258
	AN Fathers T2	22.31	[−6.74; 51.37]	0.139
	AN Fathers T3	22.92	[−7.30; 53.15]	0.143
	HC Mothers T1	21.45	[−4.88; 47.79]	0.117
	HC Mothers T2	21.21	[−5.49; 47.91]	0.126
	HC Mothers T3	22.39	[−4.81; 49.59]	0.114
	HC Fathers T1	25.47	[−1.81; 52.75]	0.074
	HC Fathers T2	23.24	[−4.45; 50.93]	0.107
	HC Fathers T3	22.30	[−5.83; 50.43]	0.127
	BSI: GSI score	3.37	[−3.09; 9.83]	0.311
	FAPAN	0.16	[−0.08; 0.41]	0.197
	CBCL sum score	0.23	[0.05; 0.41]	0.018
	Age (parents)	−0.24	[−0.78; 0.30]	0.392
	In relationship (yes vs. no)	6.10	[−0.47; 12.67]	0.074
	Age (child)	−0.44	[−2.11; 1.22]	0.603
	Weight	0.26	[−0.06; 0.59]	0.121
	EDI‐2 sum score (self)	0.05	[0.00; 0.10]	0.066
Total stress scale	AN Mothers T1	24.98	[−34.95; 84.90]	0.417
	AN Mothers T2	21.91	[−39.05; 82.87]	0.484
	AN Mothers T3	23.02	[−39.20; 85.25]	0.471
	AN Fathers T1	11.54	[−50.53; 73.61]	0.717
	AN Fathers T2	24.20	[−38.51; 86.92]	0.453
	AN Fathers T3	30.48	[−34.55; 95.51]	0.362
	HC Mothers T1	29.52	[−27.25; 86.30]	0.313
	HC Mothers T2	34.32	[−23.20; 91.84]	0.248
	HC Mothers T3	36.23	[−22.34; 94.79]	0.231
	HC Fathers T1	31.45	[−27.45; 90.35]	0.300
	HC Fathers T2	34.63	[−25.14; 94.41]	0.261
	HC Fathers T3	15.13	[−45.60; 75.87]	0.627
	BSI: GSI score	26.82	[12.49; 41.14]	0.001
	FAPAN	0.46	[−0.05; 0.98]	0.081
	CBCL sum score	0.19	[−0.22; 0.60]	0.368
	Age (parents)	0.45	[−0.73; 1.63]	0.456
	In relationship (yes vs. no)	14.28	[−0.31; 28.87]	0.059
	Age (child)	−1.90	[−5.47; 1.67]	0.301
	Weight	0.64	[−0.06; 1.33]	0.079
	EDI‐2 sum score (self)	0.11	[0.00; 0.21]	0.044

Abbreviations: AN, Anorexia nervosa group; HC, Healthy children; PSI, Parenting stress index.

### 3.3 Parenting Stress Compared Between Mothers and Fathers

Comparing mothers and fathers directly in our main analysis accounting for the mentioned covariates provided evidence that mothers and fathers in the AN group differed regarding parenting stress (parent domain: *p* = 0.026; child domain: *p* = 0.020; total stress: *p* = 0.063; robustness check: *p* = 0.032, *p* = 0.040, and *p* = 0.103). This effect was mainly driven by the difference at admission (T1), where mothers had significantly higher stress levels than fathers (parent domain: *p* = 0.013; child domain: *p* = 0.007; total stress: *p* = 0.036; robustness check: *p* = 0.019, *p* = 0.012, and *p* = 0.145). There is no evidence of significant differences between mothers and fathers in the AN group at discharge or follow‐up any more. For the HC group, evidence of significant differences between mothers and fathers was only found for the total stress score at T3, where fathers had a significantly lower stress level. This is, however, based on observations of only 8 fathers and 11 mothers in the HC group at follow‐up (T3).

### 3.4 Influencing Factors on Parenting Stress of Parents of Adolescents

The own psychological symptoms (BSI) were highly relevant in the LMM main analysis for the parenting stress: higher BSI scores were associated with much higher parenting stress in the parent domain (regression coefficient: 19.42 [95% CI: 8.83; 20.01]; *p* = 0.001) and total stress scale (regression coefficient: 26.82 [95% CI: 12.49; 41.14]; *p* = 0.001). This was also found in the sensitivity analyses for mothers and fathers separately, though for mothers it was not statistically significant (parent domain: *p* = 0.071; total stress scale: *p* = 0.063). In single models, some other variables reached statistical significance (joint model: EDI‐2 sum score, child weight, CBCL sum score; mothers: FAPAN; fathers: relationship status, child weight); however, considering the regression coefficients showed that changes in each variable were associated with only a very slight and not clinically relevant change in parental stress. For this reason, these results were not interpreted. The effect of the relationship status on the fathers' stress was not interpreted due to the small number of single fathers (*n* = 2) included in the LMMs. For complete results of the joint analysis, see Table 3; for sensitivity analyses, see Tables S1 and S2.

## 4 Discussion

In the current study, the course of parenting stress in parents of adolescents with AN during and after inpatient treatment was compared to that of parents of healthy adolescents, looking at mothers and fathers separately. According to our hypothesis, we identified higher parenting stress in mothers of adolescents with AN compared to those of healthy adolescents; in contrast, for fathers of adolescents with AN, lower parenting stress was found compared to those of healthy adolescents at admission. Contrary to our hypothesis of a consistently declining parenting stress across treatment, we found this only for maternal parenting stress in the AN group, and instead, an increase of child‐associated parenting stress in AN fathers from admission to discharge; at and after discharge, these changes resulted in no difference between the groups any more. Comparing mothers and fathers within the AN group directly showed that mothers had higher stress scores than fathers, especially at admission. Partly in line with our hypothesis, only the own parental psychological symptoms were relevant for parenting stress in both mothers and fathers.

In accordance with former studies, we identified higher parenting stress and more psychological symptoms in mothers of adolescents with AN compared to HC at admission (e.g., Duclos et al. 2023; Orive et al. 2013; Timko et al. 2023). Higher scores hint at the high importance of the own psychopathology of parents for parenting stress discussed later in more detail. In a direct comparison, AN mothers had higher child‐related parenting stress scores compared to fathers, especially at admission, which again supports former studies (Kyriacou et al. 2008; Martín et al. 2015). This difference might be explained by the differing roles of parents, including a greater responsibility within the family for the care of the child and child‐related issues and a higher emotional involvement in mothers (e.g., O'Sullivan et al. 2024; Van Lissa et al. 2019; Zeiler et al. 2023). Fathers of adolescents with AN reported perceiving mothers as confidants and primary caregivers to the child and themselves more as supporters of mothers (O'Sullivan et al. 2024), which might be associated with more (emotional) distance from the child's problems. When comparing working hours, mothers in our study spent more time at home than fathers and were thus probably more confronted with their children's disorder‐specific behavior, so that the associated parenting stress might be more pronounced for them: More contact time with those affected by AN was associated with more caregiver distress and more accommodation behaviors elsewhere (Anastasiadou et al. 2014; Kumar et al. 2024). Furthermore, mothers were found to see more eating symptoms in their adolescents than fathers, hinting at different perceptions, at least at the beginning of the disorder (Laporta‐Herrero and Latorre 2020). Differences between mothers and fathers at the beginning of treatment are further supported by the finding that mothers reported more fears regarding treatment engagement than fathers at pre‐treatment (Stillar et al. 2022). Interestingly and unexpectedly, fathers of the AN group showed even lower parenting stress at admission than fathers of HCs. In the context of a seriously ill child, parental behaviors and stress could develop more extremely: while mothers might take on even more responsibility resulting in even more stress, fathers might experience less stress than fathers of healthy children due to a potentially even more distant or avoidant behavior and lower involvement. This difference, which has been described here for the first time, and alternative hypotheses should be investigated and validated in future studies.

The parent‐specific effect was underlined here by the differing course of parenting stress during and after adolescents' inpatient treatment: while parenting stress decreased in mothers until discharge, it increased in fathers, resulting in an “approximation” and no group differences at discharge or follow‐up any more. This might be explained by an active involvement of both parents in the adolescents' inpatient treatment, in order to relieve mothers of their high stress and avoid the paternal perception of having a peripheral role in the treatment process (O'Sullivan et al. 2024). This was implemented in the inpatient treatment evaluated here in terms of regular parent and family appointments, family meals, and therapeutic day or weekend leaves. As a result, fathers might be confronted with the situation and their children's eating disorder specific behavior (e.g., during scheduled meals at home during therapeutic home leave) more clearly, comparable to mothers before, which in turn could increase the parenting stress of fathers in a kind of time delay. It can be speculated that the maintained parenting stress levels at the 6‐month follow‐up might further hint at mid‐term changes in family structures or potentially more balanced parenting roles, which should be investigated in future research with larger samples.

Due to the high emotional distress and the fears of being part of the adolescents' treatment, it is crucial to include the parents early in the treatment and address the very normal emotional parental reactions to the situation (Stillar et al. 2022). While fathers showed lower treatment‐engagement fears and no reduced self‐efficacy as mothers did (Stillar et al. 2022), they might be a resource for more intense treatment involvement at the beginning. For example, Zeiler et al. (2023) showed that fathers benefit from an AN‐specific parental skills training in a similar way to mothers. However, the increase in parenting stress of fathers across adolescents' treatment, which was found here to the best of the authors' knowledge for the first time, must not be overlooked, but specifically addressed. As several studies describe differences between mothers and fathers (Duclos et al. 2023; Stillar et al. 2022; Zeiler et al. 2023) services for parents should be designed and implemented in a parent‐specific way, with fathers being addressed explicitly. This could be implemented, for example, in the form of a father‐centered group intervention, as achieved in a pilot study with fathers of children with mixed psychiatric diagnoses showing positive effects on parenting stress and self‐efficacy (Mestermann et al. 2023). Thereby, in order to reach many parents, alternative formats should be designed and evaluated as for example, online modules or online group sessions (Mestermann et al. 2023; Philipp et al. 2024).

As the most important influencing factor for parenting stress in both mothers and fathers, we identified the own psychological symptoms, as previously found in AN (Duclos et al. 2023) and other diseases (Çınar et al. 2021; Roberts et al. 2021; Tomeny 2017). The cross‐sectional finding of higher parenting stress being associated with more psychological stress can be understood bidirectionally: On the one hand, parents with a higher level of distress could experience the situation of caring for an adolescent with AN as a particular burden due to their own reduced resources and be more susceptible to this. On the other hand, the parenting stress due to a child with AN could exacerbate the own emotional distress and thereby be seen as a further risk factor for mental health issues (Cohen 2000; McCormack and McCann 2015). For this reason, and based on previous studies regarding the bidirectional relationship of those phenomena (Cohen 2000; Duclos et al. 2023) and the negative association of parenting stress and children's treatment outcome (Monteleone et al. 2021; Timko et al. 2023), it is recommended to explore and address the (high) stress early. In summary, parental characteristics, especially psychological symptoms, seem to be more relevant than demographic, child, or treatment characteristics. This finding underlines the need for an explicit focus on parents from the start of and during the treatment of adolescents with AN.

On the basis of the current findings, it seems highly relevant that at the beginning of and during the course of treatment, the parental psychological symptoms should be taken seriously. A standard screening for parenting stress and parental psychological symptoms would improve the clinical routine and provide starting points for improving treatment. Offering parent‐focused treatment modules, as well as, if required, appropriate own (psychiatric) support for parents is needed during adolescents' treatment of AN. Moreover, reducing parenting stress and strengthening parental skills in dealing with the AN might be achieved by step‐down approaches after inpatient treatment, as for example, home treatment afterwards (Altdorf et al. 2022). During therapeutic leaves, in day clinic settings or home treatment, parental stress caused by their children's eating disordered behavior—which might be present to a greater extent than expected by parents after inpatient treatment—could be addressed to a greater extent than during mere outpatient treatment of the adolescent.

### 4.1 Limitations

It should be noted that, although the present study included mothers and fathers, the respective samples were small and became even smaller, especially in the longitudinal course of the AN group. This still needs to be taken more seriously because the a priori power calculation of the larger FRALANA study, in which the current data were collected and analyses were carried out, was done for the primary outcome; however, the current secondary analyses regarding parental stress are underpowered. Furthermore, with the available data, we cannot rule out that there might be a pre‐selection effect in that those parents having a particularly high level of parenting stress or psychological symptoms did not participate in the study, which might lead to fewer differences between both groups. Lastly, the PSI assesses parenting stress but not specifically with regard to AN and is more representative for parents of younger children; thus, in future studies, parenting stress should also be assessed using disorder‐specific methods. An additional expansion of the methods to include questionnaires that assess parenting behavior or parental roles, as well as objective markers such as cortisol as a neurobiological stress parameter, could further differentiate and explain the course of parental stress.

### 4.2 Conclusions

The present results point to a high level of parenting and emotional stress in parents of adolescents with AN as well as differences between mothers and fathers. The need to involve parents and address their individual stress in the treatment process can be derived, whereby fathers in particular should be considered with regard to their increase in stress during treatment found here. Future studies with larger samples implementing and evaluating gender‐specific clinical treatment modules for parents of adolescents with AN are necessary to understand and support fathers equally to the typically more researched and recognized mothers.

## Author Contributions

**V. Stonawski:** conceptualization, investigation, writing – original draft, methodology, validation, visualization, writing – review and editing, formal analysis, project administration, data curation. **J. Kutzner:** writing – original draft, formal analysis, methodology, visualization. **S. Büscher:** writing – review and editing, formal analysis, methodology, visualization. **A. Hoyer:** formal analysis, writing – review and editing, methodology, visualization. **O. Kratz:** conceptualization, resources, writing – review and editing, supervision. **S. Horndasch:** conceptualization, writing – review and editing, validation, methodology, data curation, supervision, project administration, investigation.

## Ethics Statement

Ethical approval for the study was granted by the local ethics committee of the Medical Faculty at the Friedrich‐Alexander‐University Erlangen‐Nuremberg. The study was conducted in accordance with the Declaration of Helsinki.

## Conflicts of Interest

The authors declare no conflicts of interest.

## Supporting information


**Table S1:** Results from linear mixed regression models regarding the course of parenting stress (PSI) comparing AN to HC in mothers.
**Table S2:** Results from linear mixed regression models regarding the course of parenting stress (PSI) comparing AN to HC in fathers.
**Table S3:** Results from linear mixed regression models regarding the course of parenting stress (PSI) comparing AN to HC with controlling variables selected via backward‐selection for robustness checks.
**Code S1**. R‐syntax for the estimation of the linear mixed models using the R‐package lme4.
**Code S2**. Examplary R‐syntax for a general linear hypothesis *F*‐Test, testing for differences in AN fathers vs. An mothers, using the R‐package lmerTest.

## Data Availability

The data that support the findings of this study are available from the corresponding author upon reasonable request.
