# Supplementary material for: Parenting Stress in Adolescent Anorexia Nervosa: Differential Stress in Mothers and Fathers During Inpatient Treatment
Source: Int J Eat Disord. 2025 Sep 18;58(12):2366–77. doi: 10.1002/eat.24551 (PMC12703218; doi:10.1002/eat.24551)
Supplement: Supplementary file 1 — Table S1: Results from linear mixed regression models regarding the course of parenting stress (PSI) comparing AN to HC in mothers. Table S2: Results from linear mixed regression models regarding the course of parenting stress (PSI) comparing AN to HC in fathers. Table S3: Results from linear mixed regression models regarding the course of parenting stress (PSI) comparing AN to HC with controlling variables selected via backward‐selection for robustness checks. Code S1. R‐syntax for the estimation of the linear mixed models using the R‐package lme4. Code S2. Examplary R‐syntax for a general linear hypothesis F‐Test, testing for differences in AN fathers vs. An mothers, using the R‐package lmerTest. [file EAT-58-2366-s001.pdf]

Supporting information to

Parenting stress in adolescent Anorexia nervosa: differential stress in mothers and fathers during inpatient treatment

Stonawski, V., Kutzner, J., Büscher, S., Hoyer, A., Kratz, O. & Horndasch, S.

Table S1. Results from linear mixed regression models regarding the course of parenting stress (PSI) comparing AN to HC in mothers. 2

Table S2. Results from linear mixed regression models regarding the course of parenting stress (PSI) comparing AN to HC in fathers. 4

Table S3. Results from linear mixed regression models regarding the course of parenting stress (PSI) comparing AN to HC with controlling variables selected via backward-selection for robustness checks. 6

Code S1. R-syntax for the estimation of the linear mixed models using the R-package lme4. 8

Code S2. Examplary R-syntax for a general linear hypothesis F-Test, testing for differences in AN fathers vs. An mothers, using the R-package lmerTest. 9

Table S1. Results from linear mixed regression models regarding the course of parenting stress (PSI) comparing AN to HC in mothers.

PSI Mothers	*Covariate*	*Regression coefficient*	*95%-Confidence Interval*	*p-value*
Parent domain	AN T1	61.45	[-2.76; 125.66]	.067
	AN T2	68.95	[3.72; 134.17]	.044
	AN T3	70.07	[4.05; 136.10]	.043
	HC T1	66.71	[6.53; 126.90]	.035
	HC T2	72.04	[11.30; 132.79]	.025
	HC T3	73.83	[12.18; 135.49]	.023
	BSI: GSI score	20.25	[-1.19; 41.70]	.071
	FAPAN	0.51	[0.05; 0.98]	.034
	CBCL sum score	-0.28	[-0.88; 0.32]	.365
	Age (parents)	0.42	[-0.90; 1.75]	.533
	In relationship (yes vs. no)	-1.08	[-15.08; 12.92]	.881
	Age (child)	-2.07	[-5.64; 1.51]	.263
	Weight	-0.17	[-0.91; 0.57]	.653
	EDI-2 sum score (self)	0.05	[-0.04; 0.14]	.312
Child domain	AN T1	45.38	[7.48; 83.27]	.024
	AN T2	43.62	[5.15; 82.10]	.032
	AN T3	42.68	[3.65; 81.71]	.039
	HC T1	44.00	[8.59; 79.40]	.020
	HC T2	44.07	[8.24; 79.91]	.021
	HC T3	45.37	[9.11; 81.63]	.019
	BSI: GSI score	4.67	[-7.83; 17.18]	.469
	FAPAN	0.12	[-0.22; 0.46]	.480
	CBCL sum score	0.24	[-0.11; 0.59]	.189
	Age (parents)	-0.62	[-1.39; 0.14]	.121
	In relationship (yes vs. no)	3.95	[-4.24; 12.14]	.351
	Age (child)	0.24	[-1.90; 2.37]	.828
	Weight	0.00	[-0.48; 0.47]	.997
	EDI-2 sum score (self)	0.04	[-0.02; 0.11]	.182
Total stress scale	AN T1	81.53	[5.11; 157.95]	.042
	AN T2	94.08	[16.47; 171.69]	.022
	AN T3	94.95	[16.27; 173.64]	.022
	HC T1	95.14	[23.65; 166.63]	.012
	HC T2	101.65	[29.35; 173.96]	.008
	HC T3	104.15	[30.91; 177.38]	.008

	BSI: GSI score	24.60	[-0.68; 49.89]	.063
	FAPAN	1.14	[0.48; 1.80]	.001
	CBCL sum score	-0.34	[-1.05; 0.37]	.355
	Age (parents)	0.63	[-0.93; 2.19]	.432
	In relationship (yes vs. no)	-4.87	[-21.43; 11.69]	.567
	Age (child)	-2.92	[-7.22; 1.39]	.190
	Weight	-0.10	[-1.04; 0.85]	.841
	EDI-2 sum score (self)	0.07	[-0.05; 0.20]	.256

Notes. Parenting Stress Index (PSI). AN = anorexia nervosa group, HC = healthy children.

Table S2. Results from linear mixed regression models regarding the course of parenting stress (PSI) comparing AN to HC in fathers.

PSI Fathers	*Covariate*	*Regression coefficient*	*95%-Confidence Interval*	*p-value*
Parent domain	AN T1	-15.66	[-73.81; 42.50]	.602
	AN T2	-18.94	[-77.22; 39.33]	.529
	AN T3	-10.57	[-70.02; 48.87]	.730
	HC T1	-20.86	[-75.69; 33.97]	.462
	HC T2	-16.03	[-71.39; 39.33]	.575
	HC T3	-24.74	[-80.74; 31.25]	.394
	BSI: GSI score	24.46	[11.02; 37.90]	.001
	FAPAN	0.00	[-0.46; 0.45]	.988
	CBCL sum score	-0.03	[-0.39; 0.32]	.854
	Age (parents)	0.23	[-0.92; 1.37]	.702
	In relationship (yes vs. no)	29.99	[7.21; 52.78]	.015
	Age (child)	-1.19	[-4.53; 2.16]	.493
	Weight	0.92	[0.33; 1.51]	.004
	EDI-2 sum score (self)	0.04	[-0.05; 0.13]	.374
Child domain	AN T1	-2.48	[-38.18; 33.21]	.893
	AN T2	-0.80	[-36.21; 34.62]	.965
	AN T3	1.53	[-35.12; 38.18]	.935
	HC T1	-1.36	[-34.39; 31.68]	.936
	HC T2	-3.54	[-36.97; 29.90]	.837
	HC T3	-4.32	[-38.06; 29.42]	.804
	BSI: GSI score	7.36	[-0.98; 15.71]	.094
	FAPAN	0.04	[-0.31; 0.38]	.835
	CBCL sum score	0.17	[-0.04; 0.39]	.125
	Age (parents)	0.32	[-0.35; 1.00]	.356
	In relationship (yes vs. no)	13.31	[-0.91; 27.53]	.076
	Age (child)	-1.32	[-3.35; 0.70]	.212
	Weight	0.40	[0.01; 0.78]	.051
	EDI-2 sum score (self)	0.04	[-0.03; 0.10]	.291
Total stress scale	AN T1	-27.23	[-104.25; 49.78]	.494
	AN T2	-28.23	[-104.62; 48.16]	.475
	AN T3	-17.56	[-96.64; 61.52]	.667
	HC T1	-25.08	[-96.33; 46.17]	.496
	HC T2	-21.43	[-93.55; 50.69]	.565
	HC T3	-41.95	[-114.71; 30.82]	.269

BSI: GSI score	30.08	[12.07; 48.09]	.003
FAPAN	-0.16	[-0.91; 0.59]	.676
CBCL sum score	0.20	[-0.26; 0.66]	.397
Age (parents)	0.70	[-0.75; 2.15]	.353
In relationship (yes vs. no)	43.87	[13.17; 74.57]	.008
Age (child)	-1.69	[-6.05; 2.68]	.456
Weight	0.91	[0.08; 1.75]	.039
EDI-2 sum score (self)	0.12	[-0.02; 0.26]	.109

Notes. Parenting Stress Index (PSI). AN = anorexia nervosa group, HC = healthy children.

Table S3. Results from linear mixed regression models regarding the course of parenting stress (PSI) comparing AN to HC with controlling variables selected via backward-selection for robustness checks.

PSI	*Covariate*	*Regression coefficient*	*95%-Confidence Interval*	*p-value*
Parent domain	AN Mothers T1	19.35	[-4.94; 43.64]	.121
	AN Mothers T2	16.18	[-10.78; 43.15]	.242
	AN Mothers T3	17.34	[-9.87; 44.55]	.214
	AN Fathers T1	9.05	[-16.00; 34.10]	.480
	AN Fathers T2	15.37	[-11.51; 42.25]	.265
	AN Fathers T3	20.84	[-7.37; 49.04]	.151
	HC Mothers T1	15.25	[-11.69; 42.19]	.270
	HC Mothers T2	19.35	[-8.08; 46.77]	.170
	HC Mothers T3	18.66	[-9.64; 46.97]	.200
	HC Fathers T1	14.79	[-12.53; 42.12]	.292
	HC Fathers T2	19.38	[-8.25; 47.01]	.173
	HC Fathers T3	10.89	[-17.80; 39.58]	.459
	BSI: GSI score	20.39	[11.62; 29.15]	<.001
	FAPAN	0.35	[0.05; 0.65]	.025
	In relationship (yes vs. no)	11.22	[0.39; 22.06]	.046
	Weight	0.54	[0.11; 0.97]	.017
Child domain	AN Mothers T1	24.79	[12.88; 36.69]	<.001
	AN Mothers T2	21.68	[9.97; 33.38]	<.001
	AN Mothers T3	20.88	[7.93; 33.82]	.002
	AN Fathers T1	17.57	[5.28; 29.87]	.006
	AN Fathers T2	22.08	[9.97; 34.19]	<.001
	AN Fathers T3	24.06	[10.35; 37.77]	.001
	HC Mothers T1	23.10	[14.71; 31.50]	<.001
	HC Mothers T2	22.98	[14.06; 31.90]	<.001
	HC Mothers T3	24.28	[15.20; 33.36]	<.001
	HC Fathers T1	28.10	[19.87; 36.33]	<.001
	HC Fathers T2	25.43	[16.82; 34.05]	<.001
	HC Fathers T3	24.64	[15.74; 33.54]	<.001
	CBCL sum score	0.30	[0.15; 0.45]	<.001
	EDI-2 sum score (self)	0.05	[0.01; 0.10]	.023
Total stress scale	AN Mothers T1	78.03	[60.80; 95.27]	<.001
	AN Mothers T2	82.60	[68.41; 96.78]	<.001
	AN Mothers T3	83.66	[66.95; 100.37]	<.001
	AN Fathers T1	68.28	[49.38; 87.18]	<.001
	AN Fathers T2	89.32	[75.01; 103.64]	<.001
	AN Fathers T3	94.67	[74.35; 115.00]	<.001
	HC Mothers T1	82.72	[72.17; 93.27]	<.001

	HC Mothers T2	88.54	[76.59; 100.48]	<.001
	HC Mothers T3	89.42	[76.48; 102.36]	<.001
	HC Fathers T1	88.59	[75.31; 101.87]	<.001
	HC Fathers T2	90.12	[75.43; 104.81]	<.001
	HC Fathers T3	71.89	[56.38; 87.40]	<.001
	BSI: GSI score	24.52	[12.26; 36.78]	<.001
	FAPAN	0.72	[0.25; 1.18]	.003

Notes. Parenting Stress Index (PSI). AN = anorexia nervosa group, HC = healthy children.

Code S1. R-syntax for the estimation of the linear mixed models using the R-package lme4.

```
lme4::lmer(formula = PSI_child_domain ~ 0 + I(group:parent:timepoint) +
             BSI_GSI_score + FAPAN + CBCL_sum_score + age_parents + relationship +
             age_child + weight + EDI2_sum_score + (1 + parent | ID))
lme4::lmer(formula = PSI_parent_domain ~ 0 + I(group:parent:timepoint) +
             BSI_GSI_score + FAPAN + CBCL_sum_score + age_parents + relationship +
             age_child + weight + EDI2_sum_score + (1 + parent | ID))
lme4::lmer(formula = PSI_total_stress ~ 0 + I(group:parent:timepoint) +
             BSI_GSI_score + FAPAN + CBCL_sum_score + age_parents + relationship +
             age_child + weight + EDI2_sum_score + (1 + parent | ID))
lme4::lmer(formula = PSI_child_domain ~ 0 + I(group:timepoint) +
             BSI_GSI_score + FAPAN + CBCL_sum_score + age_parents + relationship +
             age_child + weight + EDI2_sum_score + (1 | ID),
           subset = parent == "Father")
lme4::lmer(formula = PSI_parent_domain ~ 0 + I(group:timepoint) +
             BSI_GSI_score + FAPAN + CBCL_sum_score + age_parents + relationship +
             age_child + weight + EDI2_sum_score + (1 | ID),
           subset = parent == "Father")
lme4::lmer(formula = PSI_total_stress ~ 0 + I(group:timepoint) +
             BSI_GSI_score + FAPAN + CBCL_sum_score + age_parents + relationship +
             age_child + weight + EDI2_sum_score + (1 | ID),
           subset = parent == "Father")
lme4::lmer(formula = PSI_child_domain ~ 0 + I(group:timepoint) +
             BSI_GSI_score + FAPAN + CBCL_sum_score + age_parents + relationship +
             age_child + weight + EDI2_sum_score + (1 | ID),
           subset = parent == "Mother")
lme4::lmer(formula = PSI_parent_domain ~ 0 + I(group:timepoint) +
             BSI_GSI_score + FAPAN + CBCL_sum_score + age_parents + relationship +
             age_child + weight + EDI2_sum_score + (1 | ID),
           subset = parent == "Mother")
lme4::lmer(formula = PSI_total_stress ~ 0 + I(group:timepoint) +
             BSI_GSI_score + FAPAN + CBCL_sum_score + age_parents + relationship +
             age_child + weight + EDI2_sum_score + (1 | ID),
           subset = parent == "Mother")
```

Notes. The variables `group`, `parent`, and `timepoint` were included as factor variables. The variable `ID` identifies the family.

Code S2. Examplary R-syntax for a general linear hypothesis F-Test, testing for differences in AN fathers vs. An mothers, using the R-package lmerTest.

```
C_matrix <- matrix(data = c(1,0,0, -1,0,0, 0,0,0, 0,0,0, 0,0,0,0,0,0,0,0,
                            0,1,0, 0,-1,0, 0,0,0, 0,0,0, 0,0,0,0,0,0,0,0,
                            0,0,1, 0,0,-1, 0,0,0, 0,0,0, 0,0,0,0,0,0,0,0),
                   ncol = 20,
                   byrow = TRUE)
lmerTest::contest(model = lmm_model_object, L = C_matrix, joint = TRUE)
```

Notes. The object `lmm_model_object` denotes the model object created using the first command in Code S1, the estimates of which are shown in Table 3, section *child domain*.
